# Updated Recommendations for HIV Prevention with Adults and Adolescents with HIV in the United States

**Published:** 2014-12-12

A new evidence-based guideline, *Recommendations for HIV Prevention with Adults and Adolescents with HIV in the United States, 2014*, is now available online at http://stacks.cdc.gov/view/cdc/26062. This guideline was developed by CDC, the Health Resources and Services Administration (HRSA), the National Institutes of Health (NIH), and five nongovernmental organizations: the American Academy of HIV Medicine, the Association of Nurses in AIDS Care, the International Association of Providers of AIDS Care, the National Minority AIDS Council, and the Urban Coalition for HIV/AIDS Prevention Services. The recommendations are intended to advance the goals of the National HIV/AIDS Strategy for the United States: prevent new HIV infections, increase the proportion of persons with HIV who are aware of their infection, prevent HIV-related illness and death, and reduce HIV-related health disparities (1). The guideline updates and expands on the *Recommendations for Incorporating HIV Prevention into the Medical Care of Persons Living with HIV* (2) published in 2003 by CDC, HRSA, NIH, and the HIV Medicine Association of the Infectious Diseases Society of America. This updated guideline is a comprehensive compilation of new and longstanding federal recommendations about biomedical, behavioral, and structural interventions to reduce the risk for HIV transmission from persons with HIV by reducing their infectiousness and their risk for exposing others to HIV.

The guideline is directed to three main audiences: clinical providers, nonclinical providers, and staff members of health departments and HIV planning groups. It is published with three companion summaries that list the subset of recommendations for each of these three audiences (3–5). The guideline might also interest persons with HIV; partners of persons with HIV; specialists in HIV planning, service delivery, policy, and legislation; and managers of medical assistance programs, health insurance plans, and health systems that serve persons with HIV.

A companion resource library web site (available at http://www.cdc.gov/hiv/prevention/programs/pwp) includes dozens of practical decision-support tools, training aids, fact sheets, and other materials that can support implementation of these recommendations.
